# Progression of comorbidities in youth with overweight or obesity during the COVID-19 pandemic

**DOI:** 10.1186/s12887-023-04259-9

**Published:** 2023-09-19

**Authors:** Erica Wee, Ashley K. Sherman, Safa Farrukh, Mark A. Clements, Kelsee Halpin, Yun Yan

**Affiliations:** 1 Children’s Mercy Kansas City, Kansas City, MO USA; 2 University of Missouri-Kansas City, Kansas City, MO USA

**Keywords:** Comorbidities, Obesity, Youth, COVID-19 pandemic, Prediabetes

## Abstract

**Background:**

Childhood obesity rates have continued to increase with the COVID-19 pandemic. However, data are limited on the impact of increasing obesity on associated comorbidities.

**Methods:**

We evaluated the progression of overweight- or obesity-associated comorbidities by investigating change in laboratory results pre–COVID-19 pandemic and post–COVID-19 pandemic onset in youth with overweight or obesity. We defined progression of comorbidities based on increase in category rather than absolute change in value.

**Results:**

HbA1c progression was seen in 19%, and LDL cholesterol progression was seen in 26%, as defined by categories. HbA1c progression and LDL cholesterol progression were significantly correlated. HbA1c and LDL cholesterol progression were significantly associated with older age and Hispanics, respectively.

**Conclusion:**

The results indicate youths with overweight or obesity have experienced progression of comorbidities during the COVID-19 pandemic. This study emphasizes the importance of early detection of comorbidities among a high-risk pediatric population.

## Introduction

The data from the Centers for Disease Control and Prevention in 2017–2018 report a childhood obesity prevalence of 19.3%, affecting 14.1 million children and adolescents in the US [1]. Obesity is a risk factor for developing type 2 diabetes mellitus (T2D), dyslipidemia, and nonalcoholic fatty liver disease, which can persist into adulthood [2]. Therefore, it is important to focus on childhood obesity prevention, detection of associated comorbidities, and early intervention.

Lifestyle modifications including dietary modifications, physical activity, and behavioral changes are considered the first-line management of obesity [2]. However, the restrictions implemented to limit the spread of the SARS-CoV-2 virus may have limited youths from engaging in healthy lifestyle practices. School closures, virtual learning, limited physical activities, increased screen time, and inconsistent routine can cause or exacerbate obesity [3–6]. These challenges led to several published studies during the COVID-19 pandemic that showed a significant rise in the rates of childhood obesity [3–6]. However, information is scarce on the impact of increased rates of obesity and sedentary lifestyles during the COVID-19 pandemic on associated comorbidities such as T2D, dyslipidemia, and nonalcoholic fatty liver disease.

This study primarily evaluated the progression of the different overweight- or obesity-associated comorbidities by investigating the change in hemoglobin A1c (HbA1c), low-density lipoprotein (LDL) cholesterol, high-density lipoprotein (HDL) cholesterol, triglycerides (TG), and alanine transaminase (ALT) in youth with overweight or obesity during the COVID-19 pandemic. This study also further evaluated each laboratory measurements to determine any associated patient characteristics.

## Methodology

### Patients and data collection

Patients < 18 years old with overweight or obesity were included. The patients were seen in a tertiary pediatric center endocrinology clinic with multiple clinic locations throughout the states of Missouri and Kansas focused on T2D prevention. Eligible patients were identified through a search of the electronic medical records. Demographic and clinical data including body mass index (BMI) and laboratory test results (HbA1c, LDL, HDL, TG, and ALT) were collected pre–COVID-19 pandemic (June 2019 to May 2020) and post–COVID-19 pandemic onset (June 2020 to May 2021). June 2020 was chosen as the pandemic onset since the clinic resumed in-person visits at this time. For those with more than 1 clinic visit during the pre- and post-COVID-19 pandemic, the visit data closest to June 2020 were chosen to be included in the analysis. Patients with type 1 diabetes mellitus were excluded.

### BMI measurements

BMI was calculated by weight in kilograms divided by height in meters squared. BMI calculation was automated by the electronic medical records. Z-score was calculated based on Centers for Disease Control and Prevention growth charts for children and teens.

### Laboratory measurements

HbA1c was analyzed by high performance liquid chromatography (Tosoh G8 HPLC Analyzer). HDL and TG were analyzed by colorimetric assay (Ortho-Clinical Diagnostics™ VITROS™ 350 System). LDL was calculated using the formula total cholesterol – HDL – TG/5. ALT was measured by enzymatic method on the same platform as HDL and TG. HbA1c measurement was chosen as it is not affected by acute disturbance as compared to serum glucose.

### Definition of comorbidities progression

In this study, we defined progression of overweight or obesity-associated comorbidities based on increase in category rather than absolute increase (or decrease in the case of HDL) in laboratory value. Table 1 summarizes the definition of progression, which was based on the Endocrine Society Clinical Practice Guidelines for Pediatric Obesity [2].

**Table 1:** Definition of Comorbidities Progression

Category	Definition
HbA1c (%)	
1	< 5.7%
2	5.7 to 6%
3	6.1 to 6.4%
4	6.5 to 8.4%
5	> 8.4%
LDL cholesterol (mg/dL)	
1	< 110
2	110–129
3	>=130
HDL cholesterol (mg/dL)	
1	> 45
2	40–45
3	< 40
TG (age < 10 years) (mg/dL)	
1	< 75
2	75–99
3	>=100
TG (age 10+ years) (mg/dL)	
1	< 90
2	90–129
3	>=130
ALT (boys) (mg/dL)	
1	<=25
2	> 25
ALT (girls) (mg/dL)	
1	<=22
2	> 22

*HbA1c, hemoglobin A1c, LDL, low density lipoprotein, HDL, high density lipoprotein, TG, triglycerides, ALT, alanine transaminase

### Ethics approval and consent to participate

This study was approved by the Institutional Review Board of Children’s Mercy Kansas City.

### Statistical analysis

SAS version 9.4 (SAS Institute, Inc., Cary, NC) was used to analyze the data. Summary statistics of the patient characteristics and progression of comorbidities included mean and standard deviation for continuous variables and number and percentage for categorical variables. Chi-square or Fisher’s exact tests were used to evaluate progression of comorbidities and relationship with other categorical variables. T-tests were used to compare groups, and paired t-tests were used for within-group comparisons of continuous variables. Statistical significance was defined as p < 0.05.

## Results

### Patient characteristics

A total of 74 youth met inclusion criteria (Table 2). Briefly, the study population had a mean pre-pandemic age of 12.5 years and was 56.8% female, 39.2% white, and 59.2% publicly insured. The mean interval between visits was 10.7 (SD 3.7) months. The overall mean BMI z-score increased from 2.5 (37.1 kg/m2) pre-pandemic to 2.6 (39.8 kg/m2) post-pandemic, although the increase was not statistically different (p = 0.45).

**Table 2:** Summary of patient characteristics (n = 74)

Variables	(n = 74)
Pre-pandemic age, mean +/- SD in years	12.5 +/- 3
*Sex at birth, n (%)*	
Female Male	42 (56.8) 32 (43.2)
*Race/Ethnicity, n (%)*	
White Black Hispanic Multiracial	29 (39.2) 23 (31.1) 16 (21.6) 6 (8.1)
Insurance type*, n (%)	
Public Private Self-pay	42 (59.2) 27 (38.0) 2 (2.8)
Pre-pandemic BMI, mean +/- SD in kg/m2 Pre-pandemic BMI, mean +/- SD in z-score	37.1 +/- 9.4 2.5 +/- 0.5

^*^Missing 3 patient data for insurance type

### Change in comorbidities

HbA1c progression was seen in 18.7%, the same as HbA1c improvement. LDL progression was seen in 25.6% but LDL improvement in only 2.3%. HDL progression was seen in 11.1%, triglyceride progression in 20.0%, and ALT progression in 14.5%.Table 3 summarizes the percentage of progression, no change and improvement in comorbidities.

**Table 3:** Percentage of progression, no change and improvement in comorbidities

	HbA1c (n = 64)	LDL (n = 43)	HDL (n = 45)	Triglyceride (n = 45)	ALT (n = 55)
Progression	12 (18.7%)	11 (25.6%)	5 (11.1%)	9 (20.0%)	8 (14.5%)
No Change	40 (62.5%)	31 (72.1%)	26 (57.8%)	30 (66.7%)	46 (83.6%)
Improvement	12 (18.7%)	1 (2.3%)	14 (31.1%)	6 (13.3%)	1 (1.8%)

A total of 64 of the 74 patients had HbA1c measured pre– and post–COVID-19 pandemic onset. HbA1c progression was significantly seen in older youths as compared to those without HbA1c progression (p = 0.04). There was no difference in sex at birth, race, and type of insurance between those with and without HbA1c progression. There was no difference in the pre-pandemic (p = 0.16) and post-pandemic (p = 0.17) BMI for those with and without HbA1c progression. 4 youths with HbA1c progression developed diabetes during the study period. Those with HbA1c progression were more likely to have LDL progression (p = 0.03). There was no statistical difference with other laboratory measurements.

A total of 43 of the 74 patients had LDL measured pre– and post–COVID-19 pandemic onset. The mean age for those with LDL progression was older but was not statistically different from those without LDL progression. LDL progression was significantly associated with Hispanic ethnicity (p = 0.01). There was no difference in sex at birth and type of insurance between those with and without LDL progression (Table 4).

**Table 4:** Characteristics of those with progression and without progression of overweight- or obesity-associated comorbidities

	**HbA1c (n = 64)**			LDL (n = 43)			HDL (n = 45)			TG (n = 45)			ALT (n = 55)		
	**HbA1c progress**	HbA1c no progress	P-value	LDL progress	**LDL no progress**	P-value	HDL progress	HDL no progress	P-value	TG progress	TG no progress	P-value	ALT progress	ALT no progress	P-value
	(n = 12)	(n = 52)		(n = 11)	(n = 32)		(n = 5)	(n = 40)		(n = 9)	(n = 36)		(n = 8)	(n = 47)	
Pre-pandemic Age, mean +/-SD in years	14.1 ± 2.3	12.3 ± 2.7	**0.04**	13.4 ± 2	12.0 ± 3	0.17	10.4 ± 2.8	12.7 ± 2.8	0.08	11.4 ± 3.7	12.8 ± 2.6	0.21	13.2 ± 3.5	12.5 ± 3.1	0.55
Sex at birth, n (%)															
Female	9 (24.3)	28 (75.7)	0.18	6 (23.1)	20 (76.9)	0.73	2 (7.4)	25 (92.6)	0.38	7 (25.9)	20 (74.1)	0.28	5 (16.1)	26 (83.9)	1.0
Male	3 (11.1)	24 (88.9)		5 (29.4)	12 (70.6)		3 (16.7)	15 (83.3)		2 (11.1)	16 (88.9)		3 (12.5)	21 (87.5)	
Race/Ethnicity, n (%)															
Black	4 (20.0)	16 (80.0)	0.54	3 (21.4)	11 (78.6)	**0.01**	2 (14.3)	12 (85.7)	0.34	5 (35.7)	9 (64.3)	0.42	2 (10.5)	17 (89.5)	0.91
Hispanic	4 (30.8)	9 (69.2)		5 (83.3)	1 (16.7)		2 (25.0)	6 (75.0)		1 (12.5)	7 (87.5)		2 (16.7)	10 (83.3)	
Multiracial	0	6 (100)		0	4 (100)		0	4 (100)		0	4 (100)		1 (20.0)	4 (80.0)	
White	4 (16.0)	21 (84.0)		3 (15.8)	16 (84.2)		1 (5.3)	18 (94.7)		3 (15.8)	16 (84.2)		3 (15.8)	16 (84.2)	
Insurance, n (%)															
Private	5 (22.7)	17 (77.3)	0.83	4 (25.0)	12 (75.0)	1.0	3 (18.8)	13 (81.2)	0.41	3 (18.8)	13 (81.2)	1.0	3 (15.0)	17 (85.0)	1.0
Public	7 (18.4)	31 (81.6)		6 (26.1)	17 (73.9)		1 (4.0)	24 (96.0)		6 (24.0)	19 (76.0)		4 (12.9)	27 (87.1)	
Self-Pay	0	2 (100)		0	2 (100)		0	2 (100)		0	2 (100)		0	2 (100)	
Pre-pandemic BMI z-score, mean +/- SD in years	2.6 ± 0.3	2.5 ± 0.3	0.16	2.3 ± 0.5	2.6 ± 0.4	0.09	2.7 ± 0.7	2.5 ± 0.4	0.21	2.5 ± 0.3	2.5 ± 0.5	0.99	2.6 ± 0.6	2.5 ± 0.6	0.79
Post-pandemic BMI z-score, mean +/- SD in years	2.7 ± 0.3	2.5 ± 0.4	0.17	2.4 ± 0.5	2.6 ± 0.4	0.10	2.7 ± 0.6	2.5 ± 0.4	0.55	2.7 ± 0.5	2.5 ± 0.4	0.15	2.6 ± 0.4	2.6 ± 0.5	0.92
HbA1c, n(%)															
Progression	-	-	-	4 (66.7)	2 (33.3)	**0.03**	0 (0)	7 (100)	1	3 (42.9)	4 (57.1)	0.17	3 (37.5)	5 (62.5)	0.05
No Progression	-	-		6 (17.7)	28 (82.4)		4 (11.8)	30 (88.2)		6 (17.7)	28 (82.4)		3 (7.7)	36 (92.3)	

Triglyceride and HDL data were available for 45 patients. There was no difference in the characteristics of those with and without progression. ALT was evaluated in 55 youths and also without any correlation in the characteristics of those with and without progression (Table 4).

## Discussion

Several published studies have evaluated the impact of the COVID-19 pandemic lockdown on weight, BMI, and lifestyle behaviors [3–9]. In this study, we evaluated the progression of overweight- or obesity-associated comorbidities in youth. One of the most striking aspects of this current study was the use of categorization to define progression, as it provides more clinical significance than the absolute change in laboratory value.

Our study showed progression of overweight- and obesity-associated comorbidities among youth during the first year of the COVID-19 pandemic. A study of 90 children with obesity in Korea [10] during the COVID-19 lockdown showed no significant increase in mean HbA1c but significant increase of mean ALT pre- and post-pandemic. Another study conducted in Italy that included 40 pediatric patients with obesity did not find any significant difference in the laboratory parameters before and after the lockdown [10, 11]. We did not find any other studies evaluating progression of comorbidities by category.

HbA1c progression was more common in older youth, which can be explained by increased insulin resistance with puberty. On the other hand, LDL progression was more common in Hispanics compared to non-Hispanics. There was no difference in other laboratory parameters. Our results showed that youth with HbA1c progression during the pandemic were more likely to have LDL progression. Earlier studies on adult T2D by Thambiah et al. [12] and Khan [13] have shown correlation between HbA1c and LDL, wherein LDL is higher in those with poor glycemic control. The results of our study showed that even without T2D diagnosis, clinically significant progression in HbA1c in youth during the COVID-19 pandemic was associated with progression in lipid abnormalities as well. This finding was echoed in a previous study [14] of adolescents with obesity. In that study, the authors concluded that adolescents with obesity have lipid abnormalities (high LDL, TG, and low HDL) that correlate with the degree of insulin resistance.

The overall results of our study suggest that progression of overweight- or obesity-associated comorbidities among youths was common during the pandemic despite no statistically significant progression of BMI z-score in this study. However, the increase in BMI is worth noting and still concerning. It is plausible this progression is the result of lifestyle changes including decreased physical activity and increased screen time that were prevalent during the COVID-19 lockdown [7–9].

Our study has its limitations, including being a retrospective chart review and having a small sample size. Other factors such as lifestyle, food security, and family support are beyond the scope of this study and were not evaluated. Medications, if any, were not reviewed. Strengths of this study include that overweight- or obesity-associated comorbidities were objectively assessed using laboratory results and definition of progression was based on category.

## Conclusions

Our study showed progression of overweight- or obesity-associated comorbidities among youth during the COVID-19 pandemic. These findings emphasize the importance of early detection of comorbidities among a high-risk pediatric population.

## Data Availability

The dataset supporting the conclusions of this article are included within the article and available upon request from the corresponding author.

## References

[1] Ogden CL, Fryar CD, Martin CB, Freedman DS, Carroll MD, Gu Q (2020). Trends in obesity prevalence by race and hispanic origin-1999-2000 to 2017–2018. JAMA.

[2] Styne DM, Arslanian SA, Connor EL, Farooqi IS, Murad MH, Silverstein JH (2017). Pediatric obesity-assessment, treatment, and prevention: an endocrine society clinical practice guideline. J Clin Endocrinol Metab.

[3] Jenssen BP, Kelly MK, Powell M, Bouchelle Z, Mayne SL, Fiks AG. Covid-19 and changes in child obesity. Pediatrics. 2021. 10.1542/peds.2021-050123 33653879

[4] Woolford SJ, Sidell M, Li X, Else V, Young DR, Resnicow K (2021). Changes in body mass index among children and adolescents during the covid-19 pandemic. JAMA.

[5] Dyer O (2021). Obesity in us children increased at an unprecedented rate during the pandemic. BMJ.

[6] Chang TH, Chen YC, Chen WY, Chen CY, Hsu WY, Chou Y et al. Weight gain associated with covid-19 lockdown in children and adolescents: a systematic review and meta-analysis. Nutrients. 2021;13(10). 10.3390/nu13103668 PMC8540321 34684669

[7] Dunton GF, Do B, Wang SD (2020). Early effects of the covid-19 pandemic on physical activity and sedentary behavior in children living in the u.S. BMC Public Health.

[8] Yang S, Guo B, Ao L, Yang C, Zhang L, Zhou J (2020). Obesity and activity patterns before and during covid-19 lockdown among youths in china. Clin Obes.

[9] Welling MS, Abawi O, van den Eynde E, van Rossum EFC, Halberstadt J, Brandsma AE (2022). Impact of the covid-19 pandemic and related lockdown measures on lifestyle behaviors and well-being in children and adolescents with severe obesity. Obes Facts.

[10] Kim ES, Kwon Y, Choe YH, Kim MJ (2021). Covid-19-related school closing aggravate obesity and glucose intolerance in pediatric patients with obesity. Sci Rep.

[11] Kang HM, Jeong DC, Suh BK, Ahn MB (2021). The impact of the coronavirus disease-2019 pandemic on childhood obesity and vitamin d status. J Korean Med Sci.

[12] Thambiah S (2016). Relationship between dyslipidaemia and glycaemic status in patients with type 2 diabetes mellitus malaysian. J Pathol.

[13] Ahmad Khan H (2007). Clinical significance of hba1c as a marker of circulating lipids in male and female type 2 diabetic patients. Acta Diabetol.

[14] Steinberger J (1995). Relationship between insulin resistance and abnormal lipid profile in obese adolescents. J Pediatr.

